# Optimizing external advisory committee meetings of Clinical and Translational Science Awards through focused pre-review

**DOI:** 10.1017/cts.2024.575

**Published:** 2024-10-14

**Authors:** Shannon L. Casey, Elizabeth S. Burnside, Allan R. Brasier

**Affiliations:** 1 Institute for Clinical and Translational Research, School of Medicine and Public Health, University of Wisconsin-Madison, Madison, WI, USA; 2 Department of Medicine, University of Wisconsin–Madison, School of Medicine and Public Health, Madison, WI, USA; 3 University of Wisconsin Department of Radiology, University of Wisconsin–Madison, School of Medicine and Public Health, Madison, WI, USA

**Keywords:** Science of team science, external advisors, program peer review, translational science, qualitative evaluation

## Abstract

External advisory committees (EACs) are critical peer-review meetings that drive improvement at Clinical and Translational Science Award Program Hubs. Despite their ubiquity, evaluations of EAC optimization and effective implementation remain scarce. We present a two-tiered approach to optimizing EAC meetings through (1) in-depth, topically focused “pre-review” meetings comprised of external topic experts and at least one standing “full-board” EAC member, followed by (2) a traditional “full-board” EAC meeting. This approach allowed pre-review discussion of program-focused topics and specific recommendations, later delivered to the full-board for review and direction. To evaluate this approach, we interviewed 18 people who planned, administered, or attended pre-review and/or full-board meetings, including internal Hub staff, external topic experts, and standing EAC members. Thematic analysis was used to explore planning, implementation, and value of our two-tiered approach *versus* the traditional single full-board approach. Interviewees preferred the two-tiered approach, noting benefits including additional time to reflect, shared identification of strengths and challenges, and discussion of solutions to share later with the full-board. Those who attended pre-review meetings described building “*transformational*,” rather than *“transactional*,” relationships with invitees through more discussion and inter-hub sharing. That increased sharing invited more exploration, discussion, and planning of next steps toward innovation.

## Introduction

The Clinical and Translational Science Award (CTSA) Program seeks to advance translational science and effectively move biomedical research findings across the translational research spectrum. The CTSA Program has undergone improvement and adaptation since its inception in 2006, as evidenced by its transition from an “infrastructure-focused program” to a “Hub and spoke model” (Hub and spoke represent CTSA institutions and their partner institutions, respectively) [1]. That transition highlighted the importance of creating a national network to advance clinical and translational research (CTR) through the coordination and communication of individuals, communities, teams, disciplines, settings, and institutions [2–5]. In 2023, the CTSA national consortium had expanded to 64 Hubs across 50 states and the District of Columbia, requiring continuous, substantial, and actionable coordination for successful inter-Hub collaboration [6]. The success of the CTSA’s inter-Hub collaboration was demonstrated by their rapid response to the COVID-19 pandemic. For example, CTSA hubs sharing electronic health information to build the National COVID Cohort Collaborative (N3C) helped accelerate development of effective approaches to the pandemic [3,7,8]. One mechanism for improvement of inter-Hub collaboration and coordination is expert, peer-reviewed feedback provided by an interdisciplinary external advisory committee (EAC).

EAC meetings are composed of multiple experts from outside Hubs or affiliations across the consortium. These annual meetings provide Hubs with individualized feedback based on their unique cultures, priorities, partnering networks, and institutional influences and allow for assessment of project management, research, education, innovation, and knowledge transfer. Many large NIH grant programs, including U and P series, require regular annual external programmatic reviews. Typical CTSA EACs follow this model to evaluate Hub CTR performance, along with successes and challenges. The prototypic EAC, composed of experts from outside Hubs provides independent assessment, advice, and direction for Hub activities. One limitation of this approach is the insufficient time the experts have allotted to understand and discuss the complex problems faced by the Hubs, as a typical EAC meeting in the post COVID era could range from 4 – 8 h in duration. Therefore, to continuously improve this feedback mechanism, a better understanding of the underlying EAC format and processes used across Hubs is necessary.

Our Hub at the University of Wisconsin-Madison Institute for Clinical and Translational Research (ICTR) had the opportunity to reflect on ways to optimize these external reviews. This led us to wonder whether creating additional meetings in preparation for the typical EAC meeting, such as a two-tiered “pre-review” plus “full-board” meeting approach, would provide opportunities for smaller groups of more specialized experts in specific domains to provide program-specific and tailored responses to issues based on our needs, constraints, and resources. We anticipated this would help address the current and substantial limitations, allowing for more targeted discussion than what is typically allotted during an interdisciplinary full-board EAC meeting. With a desire for more tailored feedback, we piloted this two-tiered approach.

The purpose of this paper is to describe our two-tiered approach to improving our EAC structure using a series of pre-review meetings oriented to increase dialogue with external topical experts in advance of a full-board meeting. We used thematic analysis to assess the planning, implementation, and value of our two-tiered approach *versus* the typical single meeting full-board approach. The goal of the evaluation was to understand the benefits and drawbacks of each approach, to interpret and articulate any relative value of the pre-review approach, identify best practices in EAC meeting focus and format, and conceptualize how to maximize our use of this valuable resource. We hope these results support CTSA Hubs in planning and implementing personalized approaches to EAC meetings.

### Design and implementation of the two-tiered approach

**Figure 1.**
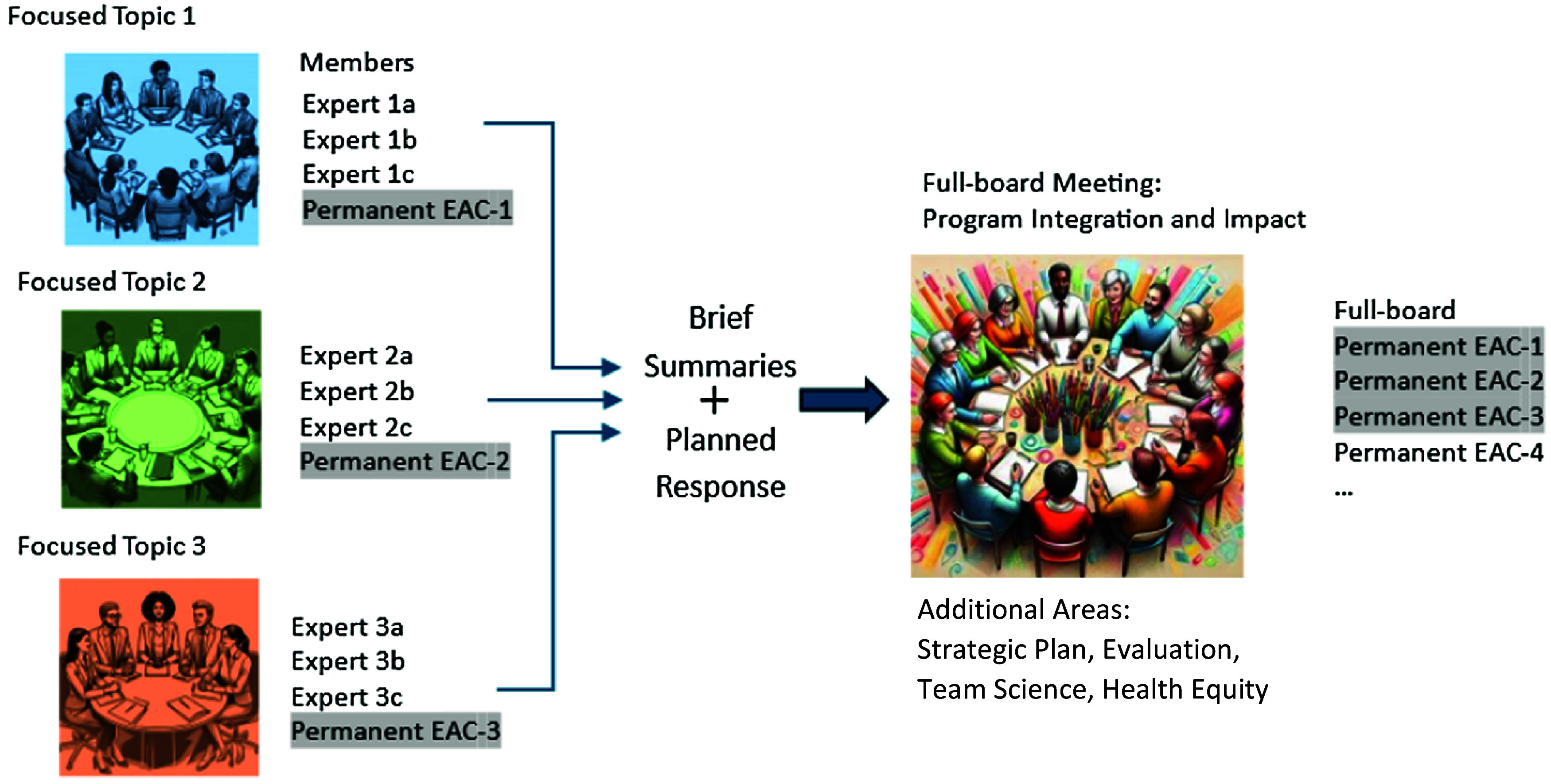
Two-tiered approach with pre-review meetings informing the full-board meeting. The schematic shows topic-focused pre-review meetings scheduled before a full-board EAC meeting. For each focused topic, program leads developed an agenda and discussion questions. Pre-review program leads selected three to five experts for pre-review meetings including at least one full-board EAC member. Arrows indicate the flow of information to the full-board EAC members. Image creation: The Adobe Acrobat Generative AI tool was accessed from Adobe Creative Cloud and used to create images, using prompt directions for “a diverse group of people surrounding a table in [blue, green, orange, multi-color];” the tool was used without modification on January 12, 2024. EAC = external advisory committee.

In 2023, our University of Wisconsin-Madison ICTR Hub implemented a two-tier approach to the annual EAC meeting, where three topic-specific pre-review meetings occurred in advance of our full-board EAC meeting (Figure 1). We anticipated that this two-tiered approach would allow for full consideration of CTSA components, like Learning Health Systems, that are unable to be fully addressed in the typical EAC model, an “everything but the kitchen sink” approach. Implementation of our approach first began with leadership, who identified three programs with current CTR priorities of relative importance that were complex enough to require substantial input and discussion. Each pre-review meeting was designed to be virtual, approximately 4 h in duration (depending on the complexity and content) and conducted one to two months before the virtual full-board 2022–2023 meeting. Meeting duration was decided by Program Leadership and determined based on the number of initiatives or themes, content, and goals.

The Program Leads were tasked with brainstorming session topics for their pre-review meeting, along with developing an agenda and a list of external advisors (not currently on the full-board EAC member panel) based on CTSA experience, domain expertise, and knowledge of timely CTSA opportunities and challenges and who could best address needs. Through conversation and consensus, Program and Executive Leaders selected and invited three external advisors from the advisor list and one or two full-board EAC members to participate in the pre-review meeting. Including a standing full-board EAC member in pre-review meetings helped maintain consistent conversation and recommendations across the pre-review and full-board meetings. In addition to explicit invitations to relevant discussants, open invitations were sent to all ICTR faculty and staff. Those involved with programs were especially encouraged to attend and learn about their own or other program priorities and hear external advisor feedback.

Next, Program Leads and topic presenters crafted brief presentations and developed relevant discussion questions (sample questions in Table 1). Program Leads were encouraged by Leadership to leave sufficient time for bi-directional dialogue, which was a meeting priority. In addition, following the structure of the typical full-board EAC meeting, time was allotted for private discussion for external advisors in the form of a virtual breakout room to integrate and prioritize feedback brought back in a final wrap-up session (see Supplemental Materials for Agenda). The week before the pre-review meeting, external committee members were sent a 1–2 page digest that included: Hub grant aims, progress on aims, meeting agenda (see supplemental materials for a sample), and proposed discussion questions.

**Table 1.** Hub pre-review meeting sample topical questions by programs sent in advance to pre-review meeting attendees

Topic area	Meeting questions
Translational endeavors/ Workforce development	How are EAC panel members incorporating principles of translational science into training programs? Specifically, what practical/operational skills are prioritized?
	How do we obtain departmental support and retain KL2 scholars over time?
	Best practices to increase cohort cohesion?
	Authentic ways to connect K and T scholars?
Learning health systems/ Implementation science	What strategies will maximize Health System and SMPH investigator engagement? Pragmatic approaches to LHS research (Quality Improvement and Clinical Trials)
	What criterion will help us prioritize LHS projects? How should we set up RFA process to select demonstration projects/use cases?
	How to expand investigator use of data-driven strategies and approaches?
Community engagement/ Networks	How do we best engage our Networks post-COVID?
	What is the best use of our multisite network?
	What value do we bring to investigators?

EACs = external advisory committees; LHS = Learning Health Systems.

Pre-review meetings were hosted by the Program Director or Lead, whose primary role was to serve as meeting chair, which included facilitating introductions, presentations, and discussions. Administrative and operations staff resolved any web-related issues in addition to serving as note-takers during the discussions and meeting wrap-up where committee recommendations were presented. Following the pre-review meetings, administrative staff sent brief, streamlined summaries of the meeting feedback and recommendations to Program Leads to review for accuracy and completeness; those summaries were then shared with pre-review meeting advisors. After those advisors revised and approved pre-review summaries, Program Leads created a brief document detailing their planned response to feedback. Pre-review meetings were scheduled at least two weeks before the full-board meeting to allow Program Leads adequate time to to craft specific action plans related to feedback. Next, all pre-review summaries and planned responses were sent to the full-board EAC members and meeting attendees along with the agenda (see supplemental materials) one week before the full-board meeting.

Then, we hosted a virtual, six-hour, full-board meeting focused primarily on internal Hub synergy and intra-Hub initiatives (e.g., Mentorship, Team Science, and ICTR Evaluation Framework). After the meeting, Program Directors, Program Leads, and Principal Investigators (PIs) co-constructed a 3-page summary, which was sent to the full-board EAC members to review for accuracy and completeness. The approved full-board meeting summary was then referred to throughout the year by ICTR members to guide the implementation of pre-review and full-board meeting feedback.

For this pilot, we held three pre-review meetings: (1) Translational Endeavors/Workforce Development that was selected because leadership needed guidance on integrating team science and translational science teaching across a range of diverse training programs; (2) Learning Health Systems (LHS) including Dissemination & Implementation (D&I) Science that was selected as a re-emphasized priority due to recent funding as a CTSA optional module with a transformative opportunity to integrate ICTR’s D&I science strengths into its LHS approach; and (3) community engagement including networks, which warranted input on the impact of COVID on clinical trials and optimal allocation of our Hub efforts to participate meaningfully with the Trial Innovation Network. All pre-review meetings, except for the private external advisor discussion, were open for all Hub employees and were recorded for future reference.

### Evaluation of the pre-review model

Three months following the full-board meeting, we evaluated this two-tiered approach *versus* the typical EAC meeting approach. We designed an evaluation that consisted of qualitative interviews with individuals who attended both the pre-review meetings and the full-board meeting, and also interviewed those who attended only one meeting. Those interviewees included CTSA administrators, PIs, Program Leads, external advisors, and full-board EAC member attendees following our 2023 meetings. Interview questions (see Supplemental Materials) centered on the relationship between the meeting format, content presented, external advisor expertise through exchange of ideas, and giving or receiving actionable feedback.

## Method

Our pre-review meeting evaluation had four phases: (1) We designed a qualitative interview; (2) we sent interview invitations to all Program Directors, PIs, Administrators, and full-board EAC members; (3) a trained interviewer conducted qualitative interviews virtually; and (4) we conducted a thematic analysis and interpretation of interview findings. Program evaluation is within the scope of our Hub Institutional Review Board (IRB); we did not pursue additional IRB approval because this effort evaluated our internal process and was not considered a research study.

### Qualitative interview

Qualitative interview questions were designed by our leadership team and Director of Evaluation (see supplement) and were centered on planning, attending, and learning from typical full-board EAC meetings, compared to this two-tiered approach. Questions assessed the benefits and drawbacks of the two-tiered approach and how topical content, dialogue between members, and member expertise influenced meeting outcomes. A trained qualitative interviewer with mixed-method experience in university settings conducted semi-structured interviews to engage flexibly with interviewees. Most interviewees participated as a planner, an observer, or an external advisor, either at our Hub or at other Hubs, at various times throughout their careers. The interviewer probed for specific experience with our EAC meetings, though often interviewees commented on their experiences at their own or other Hub meetings. We used purposive sampling to maximize responses from our network of experienced individuals [9], meaning any person who attended our pre-review or full-board meeting(s) and *also* played a role as a Program Lead, PI, Administrator, or external EAC member was invited to participate. Only four interviewees attended only one meeting, split between two interviewees at a pre-review meetings only and two interviews at the full-board meeting only. Of the 19 invited participants, 18 participated in recorded, 30-min one-on-one, virtual interviews. Interviewees included internal Hub PIs or Program Leads (*n* = 9), external advisors at the pre-review and/or full-board meeting (*n* = 7), and Administrators (*n* = 2). Interviewees came from three health science schools (Medicine, Nursing, and Pharmacy) and 2 units (Affiliated Health System and clinical trials support infrastructure). Interviewees identified themselves as Directors and Associate Directors of Hubs, tenured Full and Associate Professors, and Scientists, and 11 used she/her pronouns and seven used he/him pronouns. Only one interviewee deferred due to family illness and the interview was later cancelled due to reaching thematic saturation.

### Data analysis

Virtual interviews allowed for verbatim transcripts that were verified by the interviewer watching, correcting, and confirming accuracy. A trained qualitative researcher engaged reflexive thematic analysis [10,11] to develop themes that were conceptualized as patterns of shared meaning. Our goal was to reach thematic saturation (i.e., when no new or additional information is observed) [12,13] to ensure critical considerations were identified. Methodology experts express the importance of thematic saturation as a criterion for judging the number of interviews required to explore health science [12]. Saturation could be reached in as little as five or as many as 15 interviews for phenomenological studies, with some studies finding saturation within 12 interviews [12]. In this study where interviewees are experts, a smaller number (e.g., *n* = 7) [14]) is sufficient to correctly classify data. All interviews were analyzed. Analytic foreclosure through thematic coding [11] was only after multiple interviews had been read multiple times. Conversations between ICTR leadership and key personnel engaged in planning, executing, and participating in EAC meetings generated consensus on the qualitative findings and their implications.

## Results

### Typical EAC meeting considerations

We parsed out best practices for planning and executing a typical full-board EAC meeting compared to specific response to our two-tiered approach. That led to a general list of typical full-board EAC meeting recommendations (see Table 2), though that was not our primary intent. Interviewees described typical EAC meetings as critical for setting Hub priorities and best practices. Many interviewees articulated: “*There is no perfect EAC meeting approach or perfect hub*” and “*no meeting can possibly cover everything*.” Interviewees mentioned the history of EAC meetings for driving innovation, as evidenced here: “*We advance academic medicine by not staying in our silos. CTSAs were created to break down barriers… more innovation leads to acceptance of more innovation*.”

**Table 2.** General recommendations and pitfalls for planning EAC meetings with exemplary quotes

Recommendation	Exemplary quotes
Provide plans for next steps, invite constructive commentary and act on it	*Here are the things we have been trying and (that have) gotten us to where we want to go, and here is where we want to go, do you have thoughts about what we can do to get there?*
Seek breakthrough, “outside-the-box” thinking by prioritizing discussion over presentation	*The way we will advance the culture of academic medicine is by busting out of traditional lanes, going beyond checking a box, through education and dialogue at the cutting-edge.*
Maximize input through more transformational, rather than transactional, collaborative relationships	*Stellar meetings are where Hubs have travelled a long road to get to where they want to go. I have traveled the journey with them. That is the real joy. I get to see them develop a program out of connections with very close colleagues that have similar interests and passions.* *These are incredibly rich relationships, which are developed over time and provide mutual, symbiotic, relationships. There is reciprocity, connectedness, forward momentum.*
In addition to strengths, discuss challenges, or barriers	*There is tension. CTSAs don’t want to air their dirty laundry, but they need help with the laundry. They want to show their great stuff, but they don’t need help on stuff they do well. Parading only accomplishments prevents getting meaningful input to problem solve.*
Collaborate on strengths and opportunities with curiosity and humility	*I’m learning how you solve specific problems, not just those that exist at other institutions, but that I probably have too. I get to build on my work, have additional people to collaborate with, and more creativity with different thinking and different personalities, all that good stuff.*
Engage diverse perspectives and voices	*Are multiple perspectives represented? Do we have adequate representation? I specifically say we need people who are end users of our work, whether that is community or industry or clinicians… to help us think about what we are missing. Their positionality is different enough to be complimentary or additive. If we become too narrowly focused, we miss opportunities, we might just get it wrong.* *We have gone from being very PI heavy, we know what they will say, and it can be redundant, to having more integrated knowledge from technical specialists, community members, team science, or evaluation… a PI doesn’t always know what’s going on based on positionality.*
Bring in real-world implications	*Being focused uniquely on process deprives us of a better understanding of the impact that each of our pieces have, and then what kind of meaning that has on the real world.*
***PITFALLS***	
Being inauthentic	*EACs can be pro forma. PI’s check off a box. They give you a little chance to speak, but they want a memo saying it’s done, and then [EAC members] bless what we’re doing…that’s a waste of time.*
Not allowing adequate time for dialogue	*When you hear information, you process it and react. When you engage in dialogue, however, you process that information in a different way. You can go from knowledge to understanding and that’s a huge difference.*
Not responding to previous feedback	*I get really troubled when there is abject unresponsiveness to critiques year after year.*
Presenting only strengths	*If it only serves to present strengths, it’s not an opportunity to educate people because what is truly innovative gets eclipsed by presentation of what is already known and expected. That prioritizes checking a box over deep discussion of new ideas.*

CTSA = Clinical and Translational Science Award; EACs = external advisory committees.

There was universal agreement that both the pre-review and full-board approaches provided tremendous opportunities for reflection, discussion, summation, and planning.

### Evaluation of the two-tiered approach

Thematic analysis revealed universally positive reactions to the two-tiered approach. All Program Directors identified that the pre-review meetings were particularly valuable for planning, revising, and prioritizing interventions. Interviewees highlighted multiple factors that could drive the choice between a two-tiered approach or a full-board only approach, which included the need for feedback, Hub-specific challenges, committee member representation, timing of the meeting, and available resources. Interviewees confirmed that the value of EAC member feedback is a direct result of bi-directional dialogue, external advisor expertise, and requests for guidance. One interviewee summarized: *EAC meetings are sometimes presented as a blanket solution for getting feedback, but we surely don’t have blanket problems. There are pieces that are very specific, very situational, problems*.” A few interviewees commented that explicit preparation, particularly for pre-review meetings, requires Hub alignment on pressing issues, priorities, and opportunity creation.

*Two-tiered approach benefits/drawbacks.* There was universal agreement, including from those who attended only the full-board meeting, that the two-tiered approach benefited the EAC process: “*If the goal of any EAC is to get external input into education programs, or other specific areas, this new pre-meeting format is optimal.*” Interviewees described pre-review meetings as “*deeper dives*” with more discussion, more granularity, more frankness, and more feedback from external content experts facing similar challenges. Primary benefits were described as “*more nuanced feedback.”* More time for dialogue and connection allowed for developing relationships over time, thus relationships felt more transformational than transactional. While most interviewees acknowledged the benefit of more specific feedback, we did not ask and interviewees did not comment upon the kinds of feedback (e.g. strategic direction, program insights, instruction) that were afforded by pre-review meetings compared to full-board meetings.

Interviewees commented that internal meetings, held to prepare for pre-review meetings, were particularly valuable. One interviewee said: *“It’s an opportunity for matrixed integration, we get to hear from our colleagues across components. That sparks me to think in new ways about my own component.”* One interviewee described that preparation as: “*It’s not just a dress rehearsal… We have deeper understanding of where we align as a Hub, not just presenting in our own silos, in advance of meeting any* [full-board] *experts*.” Interviewees also commented that more people were available to help plan meetings, resulting in fewer demands to Hub and Program leadership, stating:

*“As a Hub PI, administrative burden is less. We had greater involvement of leaders planning pre-meetings. They set agendas and identified pertinent questions. There was greater intellectual input and thought behind pre-meetings from program leaders.”*

Interviewees described pre-review meetings as preventing “*hurried*” or “*superficial*” full-board meetings because there was more time for topic-focused dialogue. Some described the cost of rushed meetings as too high, commenting: “*Truncated meetings limit talk about values and what’s important. With multiple day meetings pre-COVID, we used to debate things. Those debates made me think about broad versus deep coverage, and better make the case for our contributions.”* Another interviewee identified an ethical cost, stating: “*without extended conversation, we live in a world of confirmation bias.*” Most interviewees agreed pre-review meetings led to improved full-board meetings, especially when results were shared in advance with full-board attendees and discussed within a larger context, illustrated here:

*“At our pre-meeting we discussed cutting edge ideas brought to the large group… Some of that is educating* [full-board members] *about areas they may not know as well. Asking people to step out of their lane and look holistically at intersections… that allows for truly innovative conversations.”*

Leadership identified that the compressed turn-around time between pre-review external reviewer feedback and Program Leader presentations to the full-board of concrete plans for next steps, led to accelerated transformation of feedback into action.

Although most were favorable toward the two-tiered approach, interviewees did mention drawbacks. One notable drawback was not having all pre-review meeting attendees at the full-board meeting: “*We end up with a Venn diagram of unique content experts, some who attend one meeting and others who attend both meetings. The full-day attendees don’t get access to it all.”* Those who attended only the full-board meeting explicitly identified the value of pre-review meeting feedback summaries, presentations, and discussion. They requested more time than one-week to review those summaries. Interviewees commented that the intentional overlap between pre-review and full-board attendees, in addition to summaries, were critically important to more seamless integration. Other concerns included the resources needed to support multi-day virtual meetings and “*information-loss*” associated with delays between meetings.

*Pre-review meeting focus.* A focus on approaches to, and challenges of, translational science were described as *“where the learning happens*.” Interviewees supported reviewing areas most consistent with Hub identity: “*We align content with what we want our Hub image to be, by that I mean we send messages to other CTSA leaders about who and what we are.”* Interviewees recommended that pre-review meetings should address both content and process, ideally focusing on urgent and critical needs: *“We get opinions on what direction we should follow to overcome specific challenges. There is no ambiguity. EAC members share their own experience trying to accomplish similar goals.”* Limiting the focus to only areas of strength was perceived as: “*a waste of time, no one needs help with what they are doing well.”* Challenges faced by large, often distributed, CTSA Hubs were conceptualized as normative.

Rather than pro-forma or inauthentic meetings, interviewees described most benefit out of pre-review meetings that generated solutions with authentic, engaged listening, conversation, and collective innovation. There was near universal consensus for balancing discussion and presentation time, as reflected by this comment: “*A presentation with 5 minutes to ask questions is … tedious, unproductive, unhelpful… Too much talking at us. Please maximize discussion time.*” In addition, interviewees recommended that politically charged or contentious material, or material that warrants longer or confidential discussion, be addressed in more discrete venues such as sidebar conversations that happen during in-person meetings.

*Pre-review meeting format.* Although no universal consensus emerged on the “optimal” format for full-board meetings, most interviewees reported that the virtual format was effective for pre-review meetings. Considerations for meeting format included grant timing (e.g., renewal cycle, reporting), whether a single or multiple grants are under review, familiarity with EAC members, the necessity of feedback, financial resources for travel, and urgency of anticipated change. Multiple interviewees expressed the importance of intentional selection of in-person, virtual, or hybrid meetings. The preference for in-person full-board meetings was based on the desire to build member collegiality, facilitate difficult conversations in more private settings, and recognize subtle nuances in facial expressions and behaviors. One interviewee acknowledged that meeting other EAC members in-person on occasion or in conjunction with larger meetings had value: “*A lot of my national colleagues I met on EACs. It’s a blessed thing… Relationships that oftentimes were cultivated originally in person, are sustained now through the virtual environment.”* Although most preferred in-person meetings, *o*ne interviewee summarized drawbacks, saying:

*“If it involves a lot of travel or in person, I step away… Travel can be an extra 10 to 12 hours and a night in a hotel. Flights get cancelled. You lose work time because [travel time] isn’t usable time.”*

Interviewees reported the ease of virtual meetings for pre-review, with benefits including scheduling convenience, expert or EAC member availability, and recording meetings for later viewing. Yet, some identified limitations that included confirmation bias, described by one advisor as

*“Zoom is highly efficient… but it’s horrible. You don’t have richness or opportunity to discuss problem areas over lunch. EAC members don’t spend evenings talking about how to make a program the best it can be.”*

There was universal consensus against hybrid meetings, summarized by this comment: “*It’s like teaching hybrid. Recently a Hub had their team in a room and external advisors were virtual. The speaker picked up every background noise. It was very challenging to be distracted. It’s just too difficult for everyone to make equal contributions.”*

Interviewees gave specific options for determining format, including: (1) selecting committee members before format and engaging committee members in determining format; (2) identifying whether meeting in-person is more important than a particular constituency of members; and (3) maximizing transformational relationships between meeting participants. If costs are a limiting factor in deciding meeting format, interviewees suggested decreasing the number of full-board EAC members traveling to an in-person meeting and increasing the numbers of pre-review meeting experts invited to participate virtually.

## Discussion

Our goal was to identify the benefits and drawbacks of a two-tiered approach with pre-meetings in addition to a full-board EAC meeting *versus* a typical full-board EAC meeting approach to foster inter-hub knowledge sharing and continuous improvement to advance CTR. We sought explicit feedback relating to the pre-review meeting’s focus and its optimal format. The two-tiered approach provided an effective method of gathering topic-specific expert feedback from external advisors via a highly focused, interactive half-day (or less) virtual gathering. Interviewees, nearly all of whom attended a pre-review meeting, were highly favorable toward this approach and supported its use on occasion, if not annually. Benefits were wide ranging and highlighted the critical importance of member dialogue and content expertise for tailoring feedback, advancing innovation, and minimizing confirmation bias.

There was universal consensus that pre-review meetings increased productivity in full-board EAC meetings due to several factors: an increase in inter-hub and intra-hub collaboration, specific focus on key problems, planning for next steps in advance of the full-board EAC meeting, facilitating innovation, and creating an environment to discuss challenge. With regard to focus, interviewees recommended that pre-review meetings focus on timely issues and areas most in need of development. While many typical full-board EAC meeting considerations were identified (Table 2), it was clear that the value of external advisor feedback related directly to the pre-review meeting content, bi-directional dialogue, and committee member expertise. With regard to format, while most preferred in-person meetings, there was a general understanding that virtual meetings are expedient and provide opportunity for exchange of ideas with experts who may not be available to attend a meeting in-person. Overall, interviewees reported that pre-review meetings facilitated more transformational, rather than transactional, relationships because of the intentional internal planning, specific meeting times for interaction, and focused relevant exchange of ideas in one’s areas of expertise during the meetings.

With the benefit of hindsight, our Hub is now in 2024, able to recognize areas where implementation of our initiatives was influenced by feedback received in 2023 during both pre-review and full-board meetings. Though evaluating types of feedback (e.g., general or specific guidance) was outside the scope of our pre-review evaluation, we received feedback on strategic direction (e.g., carefully monitor and evaluate resource utilization to build and prune capacity; better articulate the connection between components), large-scale programmatic considerations (e.g., use shared mentor recruitment approaches across programs), and specific ideas for implementation (e.g., increase interaction between KL2 and TL1 learners). Additionally, feedback provided during the meetings allowed for execution of meaningful program decisions. Specifically, pre-review meetings focused on interventions (e.g., building collaborative opportunities between KL2 and TL1 learners; expanding use of big data enclaves), while the full-board meeting facilitated higher-level recommendations (e.g., increasing interdisciplinary science with partner schools; integrating inclusivity at all levels).

### Lessons learned

We learned important lessons for implementing this approach and planning future meetings (Table 2). We intend to maximize the outcomes of a multi-day process through:

- Careful selection of pre-review topics, focusing on programs experiencing challenges that require detailed discussion or input;
- Assuring pre-review and full-board meeting topics are complementary and avoid content duplication;
- Providing streamlined pre-review meeting summaries with recommendations and action plans to full-board attendees (e.g. executive summaries, infographics) at least two weeks in advance;
- Focusing the full-board meeting on the Hub’s response to the pre-review feedback and intra-hub program integration and evaluation.

## Limitations

Our evaluation benefited from interviewing a diverse group of experienced CTR professionals. That said, our results reflect the views of a small group of interviewees at a single point in time. Interviewees are likely biased, with positive perceptions of our Hub, as approximately half of internal interviewees were funded or employed by, or somehow professionally affiliated with our Hub. There was no tangible benefit, however, to any interviewee advocating for or against our approach. All understood that our goal was to identify honestly, confidentially, though not anonymously, the benefits and drawbacks of a two-tiered approach. We recognize that annual pre-review meetings may not be possible for a CTSA Hub. The size of our Hub and our institutional support allowed for three pre-review meetings during the first year of our competitive renewal. Though we did not test other structures, smaller or more limited Hubs may consider a single pre-review meeting in a primary area or schedule a pre-review meeting only occasionally in advance of full-board meetings. We hope these findings generate additional conversation and reflection that maximizes the value of EAC meetings for both individual Hubs and the CTSA consortium.

## Conclusion

The EAC process contributes to well-documented CTSA program successes in advancing science and the health of communities through the coordination of multi-institutional collaborative efforts [3]. Our findings support strong consideration of the two-tiered approach. While interviews did not yield generalizable statements with exacting pre-review directions, there is value in identifying primary considerations for the meeting focus and format. Interviewees, all experts in CTSA activities, surfaced meaningful consequences associated with inauthentic or hurried EAC member participation. Notably, the two-tiered approach facilitated opportunities for diverse content experts to brainstorm challenges and solutions, allowing for more discussion of innovation to plan for next steps and hopefully enhance communication of our value to our CTSA peers. Those conversations benefited our Hub members and external advisors, who collectively benefit from opportunity generation across diverse challenges within the general principles of translational science. This two-tiered approach increased inter-hub learning, in turn helping our hub efficiently, ethically, and responsibly invest funds to improve our effectiveness across a multiplicity of programs and disciplines.

## Supporting information

Casey et al. supplementary material Casey et al. supplementary material

## Supplementary material

The supplementary material for this article can be found at https://doi.org/10.1017/cts.2024.575.
