# Supplementary material for: Optimizing external advisory committee meetings of Clinical and Translational Science Awards through focused pre-review
Source: J Clin Transl Sci. 2024 Oct 14;8(1):e162. doi: 10.1017/cts.2024.575 (PMC11557274; doi:10.1017/cts.2024.575)
Supplement: Casey et al. supplementary material [file S2059866124005752sup001.docx]

**SUPPLEMENTAL MATERIALS**

**JCTS 2024-0063.R1:** ***Optimizing External Advisory Committee Meetings of Clinical and Translational Science Awards through Focused Pre-Review***

**Interview Protocol:**

WARM UP: Thinking back to our WFD/LHS/CE pre-review meeting, followed by the all-EAC/B meeting, in a sentence or two, what about ICTR’s EAC/B meetings stand out or do you remember most?

1. How do you advise your own hub or other hubs you advise about conduct of EAC/B meetings based on your experience?
2. The format of the meeting approach (*one day vs multiple days*), specific attendance, and content interacts with dialogue and feedback at a pre-review meeting versus an “all-EAC/B” meeting. What considerations do you make about scheduling pre-review meetings versus scheduling a single meeting?
   1. Thinking about content and your area of expertise, what are the benefits/drawbacks of meeting multiple days? What considerations do you make regarding content?
   2. Thinking about dialogue and your ability to ask questions or provide feedback, what are the benefits /drawbacks of meeting multiple days?  What considerations do you make as you plan for dialogue and feedback?
   3. Thinking about the time commitment relative to content and dialogue, what are the benefits/drawbacks of multiple days? What considerations do you make when planning how to use the time?
3. How would you describe the benefits and drawbacks of this pre-review meeting structure to an NCATS colleague or Program Officer? How would you describe the benefits and drawbacks of a one-day all-EAC/B meeting structure?
4. Anything else you wish to add about your ICTR EAC/B meeting experience?

NOTE: CE = Community engagement; EAC/B = External Advisory Committee/Board, ICTR = Institute for Clinical and Translational Research, LHS = Learning Health Systems, NCATS = National Center for Advancing Translational Science, WFD = Workforce Development.

**PRE-REVIEW EAC MEETING AGENDA**

**ICTR Pre-Review External Advisory Committee Meeting**

**Learning Health Systems // Implementation Science**

DATE | 1:00 pm–5:00 pm Central Time

Zoom: Link

| **Topic** | **Presenters** | **Time (CT)** |
| --- | --- | --- |
| **Welcome, Introductions, & Meeting Goals**  • Background and review of focus areas | Hub PIs | 1:00 to 1:15pm |
| **LHS Overview** | LHS Lead & Team | 1:15 to 1:45pm |
| - *LHS Components* - *Projects Underway* - *New Faculty* - *Discussion* | *LHS Lead* | *30 minutes* |
| **D&I Launchpad Partnership** | D&I Lead & Team | 1:45 to 2:15pm |
| - *D&I Components* - *Falls Project/Human Factors Design* - *Discussion* | *D&I Lead*  *Project Lead(s)* | *30 minutes* |
| **BREAK** |  | 2:15 to 2:30pm |
| **LHS Focus Area A:** Strategies for Health System and SMPH Investigator Engagement | Informatics PI | 2:30 to 3:10pm |
| - *Pragmatic approaches to LHS Research (QI & Clinical Trials)* - *Discussion* |  | *40 minutes* |
| **LHS Focus Area B:** Prioritization of LHS Projects-Demonstration Projects/Use Cases, RFA Process | LHS & Protocol Development Leads | 3:10 to 3:50pm |
| - *Demonstration Projects/Use Cases, RFA Process* - *Discussion* |  | *40 minutes* |
| **LHS Focus Area C:** Expand Investigator Use of Data-Driven Strategies & Approaches | Chief of Biomedical Informatics | 3:50 to 4:30pm |
| - *Data Driven Strategies & Approaches* - *Discussion* |  | *40 minutes* |
| **Overview of Feedback and Wrap Up** | LHS Lead | 4:30 to 5:00pm |

NOTE: CE = Community engagement; EAC/B = External Advisory Committee/Board, ICTR = Institute for Clinical and Translational Research, LHS = Learning Health Systems, NCATS = National Center for Advancing Translational Science, WFD = Workforce Development.

**FULL-BOARD EAC MEETING AGENDA**

**ICTR Full External Advisory Committee Meeting**

**DATE | 8:00 a.m.–12:00 p.m. Central Time**

Zoom: Link

| **Topic** | **Presenter(s)** | **Time (CT)** |
| --- | --- | --- |
| **Welcome, Introductions, and Meeting Goals** | Hub PIs | 8:00-8:10am |
| **EAC Pre-Meetings and Next Steps** |  | 8:10-9:10am |
| *Workforce Development –*  *Presentation & Discussion (P&D)* | *WFD Program Director* | *20 minutes* |
| *Community Engagement & Networks - P&D* | *CE Program Director* | *20 minutes* |
| *Learning Health Systems & Data Science - P&D* | *LHS Program Director* | *20 minutes* |
| **Advancing Mentorship, Team Science, and Dissemination & Implementation** |  | 9:10-10:25am |
| *Mentorship - P&D* | *Mentorship PIs* | *20 minutes* |
| *Team Science – P&D* | *Team Science Leads* | *20 minutes* |
| Break (15 mins) |  | 9:50-10:05am |
| *Dissemination & Implementation - P&D* | *D&I Leads* | *20 minutes* |
| **Cross-ICTR Evaluation**– Leveraging TSBM, Integrating Health Equity | Evaluation Lead | 10:25-10:45am |
| **Administrative Challenges** – Leadership & Capacity Building | Hub PIs | 10:45-11:15am |
| **External Advisory Committee Closed Session**  Break for all other participants | EAC Members  Private Zoom Link | 11:15-11:45am |
| **Summary of Feedback and Recommendations**  **Closing Remarks** | Hub PIs | 11:45-12:00pm |

NOTE: CE = Community engagement; EAC/B = External Advisory Committee/Board, ICTR = Institute for Clinical and Translational Research, LHS = Learning Health Systems, NCATS = National Center for Advancing Translational Science, WFD = Workforce Development.
